# Effects of Induced Mindfulness at Night on Repetitive Negative Thinking: Ecological Momentary Assessment Study

**DOI:** 10.2196/44365

**Published:** 2023-07-19

**Authors:** Amanda Sommerhoff, Thomas Ehring, Keisuke Takano

**Affiliations:** 1 Department of Psychology Ludwig-Maximilians-Universität München Munich Germany; 2 Human Informatics and Interaction Research Institute National Institute of Advanced Industrial Science and Technology (AIST) Tsukuba, Ibaraki Japan

**Keywords:** mindfulness, repetitive negative thinking, stress, daily life, ecological momentary assessment, mobile phone

## Abstract

**Background:**

Repetitive negative thinking (RNT) is a cognitive risk factor for various disorders. Although brief mindfulness-based interventions (MBIs; lasting 20-30 minutes or shorter) are effective tools to reduce RNT, the effect of a minimal (5-minute) MBI remains largely unknown.

**Objective:**

We investigated the acute changes in RNT induced by a 10-day minimal MBI (body scan before sleeping) using an ecological momentary assessment (EMA) administered during the MBI training phase. In addition, we examined longer-term effects on the postintervention and 2-month follow-up assessments for questionnaire-based RNT and psychological distress.

**Methods:**

A total of 68 participants (community sample, aged 18-55 years; n=58, 85% women) were randomly allocated to either the intervention group (n=35, 51%) or the no-training control group (n=33, 49%). Both groups completed a 10-day EMA phase of RNT, during which only the intervention group performed a daily 5-minute body scan before sleeping.

**Results:**

The intervention group showed a significantly larger reduction in questionnaire-based RNT than the control group at the follow-up assessment (for growth-curve modeling analysis [GMA], *d*_GMA_=−0.91; *P*<.001), but this effect was not observed during the EMA phase or at the postintervention assessment. Furthermore, the intervention group showed significantly larger decreases in stress both at the postintervention (*d*_GMA_=−0.78; *P*<.001) and follow-up (*d*_GMA_=−0.60; *P*<.001) assessments than the control group. We found no intervention effects on depressive and anxiety symptoms.

**Conclusions:**

A 5-minute body scan before sleeping reduces RNT and stress when continued for at least 10 days; however, the results suggest that this effect only appears with some time lag because no acute changes during and immediately after the intervention emerged for RNT.

## Introduction

### Background

It is estimated that approximately one-third of the global population is affected by mental disorders [1], which are associated with high direct and indirect costs for the individual and society [2,3]. In addition, subclinical levels of psychopathology lead to considerable dysfunction and impairment [4]. However, there is limited access to mental health services provided by mental health professionals [4], necessitating a self-management strategy to maintain and improve mental health and prevent the onset of mental disorders [5].

As a promising treatment approach for a variety of mental health outcomes, mindfulness-based interventions (MBIs) have increasingly captured the interest of the scientific community over the past 3 decades [6]. Standardized MBIs such as mindfulness-based stress reduction (MBSR) [7,8] and mindfulness-based cognitive therapy (MBCT) [9] have been established as effective prevention and treatment approaches for various mental health outcomes in clinical [10,11] and nonclinical populations [12-14]. Importantly, MBIs are shown to be effective not only in improving symptoms but also in reducing vulnerability factors such as depressive rumination and repetitive negative thinking (RNT) [15,16].

However, programs such as MBSR and MBCT require substantial time investment from practitioners [12]. Hence, brief MBIs have been developed to enhance the accessibility of MBIs.

Although brief MBIs are suitable for addressing the accessibility issue, a consensus has not yet been reached concerning how much exercise should be included in a single session and for how many days the intervention should be continued, which poses an obstacle for the widespread implementation of MBIs as a mental health tool [17,18]. A systematic review [19] found that even a minimal set of brief MBIs (ie, 5-20 minutes in duration, mostly mindfulness audios) in 1 session can have a positive effect on negative mood, anxiety, or associated factors such as rumination in healthy and clinical populations.

However, the longevity of the effects of brief MBIs is unclear [19,20], and only a few studies have examined the real-time effects of MBIs using ecological momentary assessment (EMA) designs [21-23]. In an EMA study, participants are typically asked to provide systematic self-reports of their everyday lives repeatedly during their waking hours [24]. This web-based ecologically valid assessment approach improves temporal resolution, allowing researchers to capture even brief changes in symptoms. A study administering EMA during MBSR [22] suggested that the intervention improved mindfulness skills, depression, and anxiety symptoms throughout the intervention. Interestingly, these effects emerged only in the EMA-assessed symptoms but not in the symptoms reported in the pre- and postintervention phases. Another study [23] conducted EMA during an 8-week MBI program in which participants were instructed to choose from meditations varying in length from 5 to 20 minutes. The results indicated that the effects of the MBI appeared immediately, highlighting a pronounced decline in anxiety and sleep problems and an acute increase in happiness within the first weeks of the intervention program [23]. Furthermore, the total number of minutes meditated was not a statistically significant predictor of outcomes at the 8-week assessment point [23]. There is also an EMA study [25] that targeted changes in a cognitive dysfunction (rumination) over 42 days of an MBI, which consisted of 20-minute daily meditation practice sessions. The changes in rumination seemed to follow a 3-phase pattern: during the first week of the MBI, rumination decreased rapidly; between days 10 and 30, it remained stable; and after 30 days of practice, it decreased again [25].

To expand these earlier EMA-mindfulness works, this study used EMA to examine the effect of a minimal (ie, 5-minute) MBI on a marked cognitive vulnerability factor for psychopathology, that is, RNT. We targeted RNT as the main outcome here because the theory underlying MBCT [9] highlights the role of this maladaptive thinking style as a putative mechanism of change for an MBI; specifically, it has been proposed that MBIs enhance awareness of, and disengagement from, RNT, which reduces symptoms and prevents a relapse of depression [9]. RNT is defined as excessive and repetitive thinking about one’s current concerns, problems, past experiences, or worries about the future [26]—conceptually encompassing depressive rumination and anxious worry. RNT is an important risk and maintenance factor for various mental disorders, including depression and anxiety [26], and has been a target of prevention and intervention [27-29]. Although there is no consensus concerning the mechanisms underlying an MBI to date [30], a growing body of literature suggests that alterations in RNT are a significant contributing factor to the effects of an MBI [31,32].

Although RNT is typically operationalized as a ruminative tendency (or trait factor), it has been shown that RNT has some temporal variability that may hint at how RNT-focused interventions can be implemented. An EMA study [33] found that RNT levels have a U-shaped diurnal variation—being the highest in the morning and evening. This pattern was replicated by another EMA study, which also identified individual differences owing to depressive symptoms, that is, individuals with higher levels of depressive symptoms tend to experience higher levels of ruminative thinking in the evening [34]. Evening rumination seems to be triggered by a lack of social distractors, such as interaction with others [35], and is associated with increased autonomic arousal [36] and poor sleep quality [37-39]. Therefore, the evening is a critical time window to effectively implement psychological interventions targeting RNT.

### Objectives

In this study, participants performed a 5-minute body scan before sleeping, which we expected to prevent the occurrence of, and help disengage from, RNT. As supporting evidence for an MBI at night, it has been suggested that a 6-minute mindfulness induction before sleeping improves athletes’ sleep quality (although evidence is lacking for RNT) [40]. Therefore, we aimed to provide direct evidence of the effectiveness of a minimal MBI before sleeping to reduce RNT. To establish this effect, we used a 2×3 factorial design with 2 groups (MBI group vs no-training control group) and 3 assessment points (baseline, after the intervention, and 2-month follow-up), as well as continuous EMA to capture the acute in-training changes in RNT throughout the MBI. Our primary outcome was RNT, and we also assessed psychological distress symptoms (ie, depressive, anxiety, and stress symptoms) as secondary outcomes at each assessment. First, we hypothesized that the intervention group would show a significant decrease in EMA-assessed RNT as the MBI progressed, whereas the control group would maintain their initial levels of RNT over time (hypothesis 1). Second, we hypothesized that the intervention group would show a significant reduction in questionnaire-assessed RNT from baseline to the postintervention and follow-up assessments (hypothesis 2).

## Methods

### Participants

**Figure 1:**
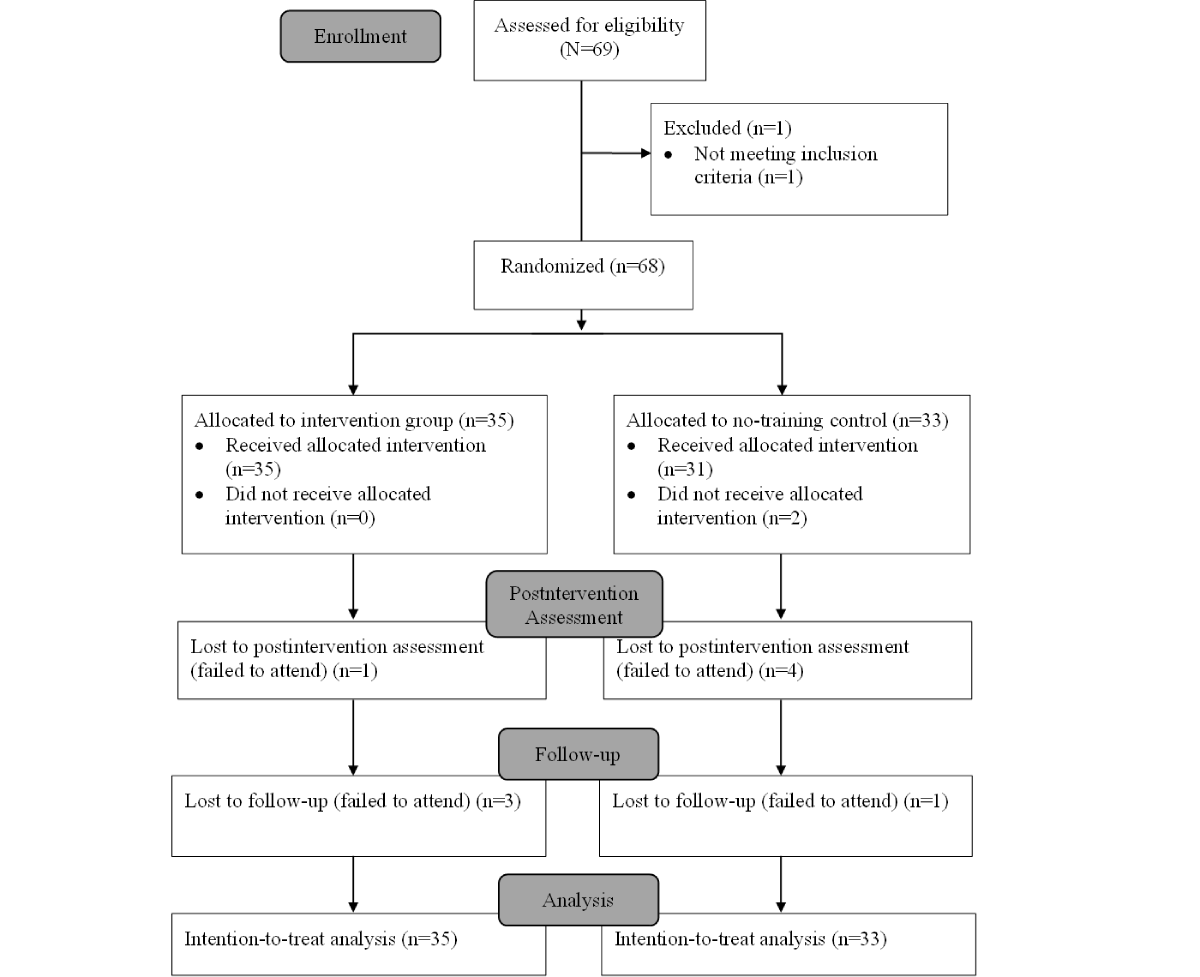
CONSORT (Consolidated Standards of Reporting Trials) flow diagram.

Participants were recruited at the university campus of Ludwig-Maximilians-Universität München (LMU Munich) and from the Munich metropolitan area on the web (Facebook and eBay Kleinanzeigen [advertisements in the personal columns]), as well as via flyers and by using the university’s email distribution list (covering graduate and undergraduate students). Participants were required to be aged at least 18 years and fluent in German and were excluded if they had practiced mindfulness daily in the past 3 months before the study [41] or had a neurological disorder or a serious disorder for which mindfulness practice may have adverse effects (cardiac function disorders, hypotension, bronchial asthma, migraines, seizure disorders such as epilepsy, attention-deficit/hyperactivity disorder, and dementia). Each participant was informed of the purpose of the study at the recruitment as follows: “The aim of this study is to investigate the effect of a short mindfulness exercise before bedtime on mood, sleep quality, and cognitions. You will be allocated to one of two groups. One group performs a short mindfulness exercise every day whereas the other group does not change anything in their everyday life.” Eligible participants (N=69) were then assessed for the inclusion criteria (Figure 1). Of these 69 participants, 1 (1%) was excluded because of an asthma condition. The remaining participants were randomly allocated to either the intervention group (35/68, 51%) or the control group (33/68, 49%) following a predetermined random allocation sequence generated by Random [42].

An a priori power analysis was conducted using G*Power [43] to determine the sample size. Querstret et al [14] reported an effect of Hedges *g*=−1.13 in the postintervention levels of rumination in nonclinical samples, which gives the required sample size as 28, with the assumptions of α=.05 and power=0.80.

### Procedure

**Figure 2:**
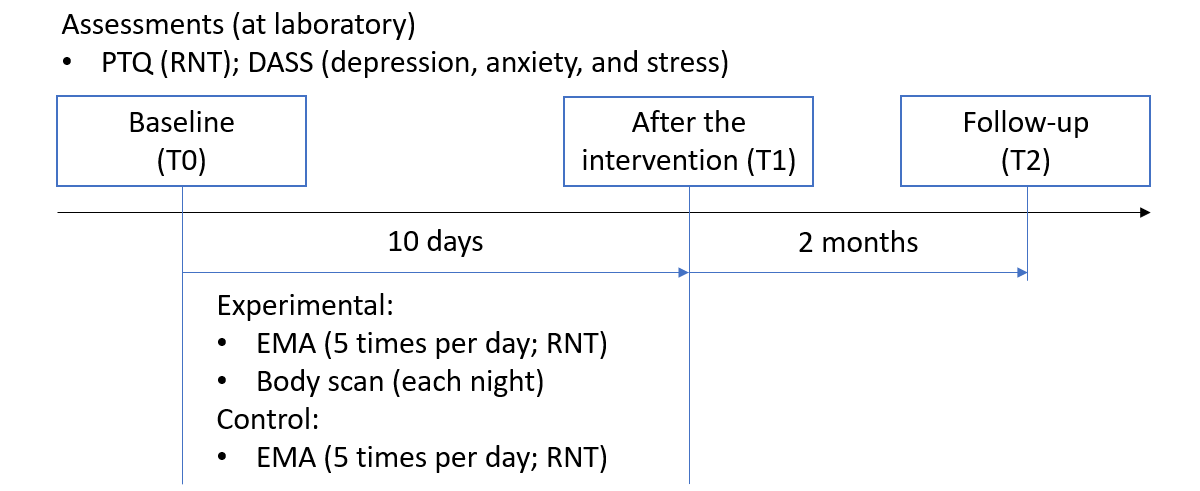
Flow of the study. DASS: Depression Anxiety Stress Scale; EMA: ecological momentary assessment; PTQ: Perseverative Thinking Questionnaire; RNT: repetitive negative thinking.

Participants attended the preintervention assessment (T0) at the laboratory of LMU Munich, where they provided written informed consent and completed questionnaires for demographics and baseline symptomatology. Note that data were collected within a larger study (Takano, K, unpublished data, October 2022) using a test battery, including other measures, that are not reported here. Participants were randomly allocated to either the intervention or the no-training control group following the randomization sequence that had been created before data collection. No blinding procedures were performed. Participants were instructed to use the EMA app (MobileQ; KU Leuven), which they had installed on their Android smartphone before the appointment (in case of technical issues or if they did not own an Android smartphone, the participants received a smartphone for the duration of the study). Upon complying with this instruction, participants read through all EMA questions, and any unclear points regarding the study procedure were resolved by an experimenter. The intervention group received further instructions for the 5-minute MBI. The day after the T0 assessment, all participants started the 10-day EMA and, if they were allocated to the intervention group, the MBI training sessions. Participants completed the same questionnaires as the ones administered at T0 on a web-based platform the day after the completion of the 10-day EMA (T1, after the intervention) and 2 months later (T2, follow-up). Furthermore, the T2 assessment included 1 question asking whether participants had performed mindfulness practice during the period between the postintervention and follow-up assessments (refer to Figure 2 for the flow of the study).

After completion of the T2 assessment, the participants were debriefed and provided with either course credit or monetary compensation. For monetary compensation, participants received €8 (US $8.8) per hour for assessments and had the chance to win 1 of 10 €20 (US $22) vouchers, depending on their compliance during the EMA. For student participants, course credit was given based on compliance during the EMA. In addition, all participants who completed both T1 and T2 assessments had the opportunity to win a €50 (US $55) voucher. The data collection started in November 2019 and ended in May 2020, and we did not change any aspect of the study protocol even after the COVID-19 pandemic.

### Ethics Approval

The ethics committee of the Department of Psychology at LMU Munich approved the study protocol (approval ID: 11_2019_Takano_b).

### MBI Practice

Participants in the intervention group performed a 5-minute guided version of the body scan derived from MBSR [7] as an MBI each night throughout the EMA. Participants were instructed to practice the body scan before going to sleep as their final activity of the day and to mindfully guide their attention from their feet to the head in a nonjudgmental manner. It has been shown that guided exercises support participants in practicing mindfulness [44], and the body scan is considered a good starting point for mindfulness practice [45]. In addition, it is one of the most popular meditation exercises among novices [46]. Participants in the control group were instructed to go about their day as usual.

### Measures

#### RNT Assessment

RNT was assessed using the 15-item Perseverative Thinking Questionnaire (PTQ) [47]. Three subscales assess RNT as a transdiagnostic process, with 9 items covering the three core characteristics of RNT: (1) repetitiveness, (2) intrusiveness, and (3) difficulties in disengaging from thoughts; for each core characteristic, 3 questions are asked concerning the unproductiveness and mental capacity captured by RNT. Items were rated on a scale ranging from 0=*never* to 4=*almost always*. The total sum score for all 15 items was computed. The PTQ is a valid measurement instrument for RNT [47]. In this study, internal consistency was excellent, with Cronbach α=.93 for the sum score and internal consistencies of Cronbach α=.92 (core characteristics), Cronbach α=.82 (unproductiveness), and Cronbach α=.84 (mental capacity) for each respective subscale.

#### Psychological Distress Symptoms

Depressive, anxiety, and stress symptoms were measured using the German short form [48] of the Depression Anxiety Stress Scale (DASS) [49] for nonclinical populations. Participants were asked to rate 21 items within the time frame of the previous week on a scale ranging from 0=*did not apply to me at all* to 3=*applied to me very much or most of the time*. The total score for each subscale was computed (depression, anxiety, and stress: 7 items each). The DASS showed good internal consistencies: Cronbach α=.83 (depression), Cronbach α=.77 (anxiety), and Cronbach α=.83 (stress).

#### EMA Method

The EMA was conducted using a smartphone app, MobileQ [50]. Participants received 5 prompts daily for 10 consecutive days. In response to each prompt, participants had to start a short survey within 90 seconds. At each prompt, participants were asked to rate 4 items adapted from the PTQ [51] on a scale ranging from 0=*not at all* to 7=*very much* concerning their current RNT: that is, “The same thoughts keep going through my mind again and again” (repetitiveness), “Thoughts come to my mind without me wanting them to” (intrusiveness), “I get stuck on certain issues and can’t move on” (uncontrollability), and “I feel disturbed by negative thoughts” (distress). The distress item was adapted from the original wording [51] to fit in the context of this study. The mean score of all 4 items was computed. In addition, the first prompt of each day included an item intended as an adherence check for the body scan, where participants were asked to indicate whether they had practiced the 5-minute body scan the previous night (compare with the study by Hülsheger et al [52]). A duration of 10 days was chosen because RNT has been shown to rapidly decrease during the first week of mindfulness practice [25]. The number of daily assessments was based on general guidelines [53]. Furthermore, a sampling design of 5 daily assessments across 10 days is recommended [51] because this sampling frequency has been shown to yield the best trade-off between participant burden and information obtained by EMA. Each EMA included items other than RNT, such as momentary levels of affect (rated on a slider ranging from 0=*unpleasant* to 100=*pleasant*). We found no significant effect of the MBI on the affect (valence) ratings, and thus we focused exclusively on RNT in the *Results* section. The first EMA prompt of each day was sent at 9 AM, and the remaining 4 prompts were scheduled at random times between 10 AM and 10 PM, each prompt separated by an average of 2 hours. The estimated time to answer all questions was 2 minutes.

### Data Analyses

Multilevel modeling was performed on the intention-to-treat sample with (restricted) maximum likelihood (ML) estimation implemented by the R packages *lme4* [54] and *lmerTest* [55]. This analytic approach has some advantages in modeling the random effect structure and handling missing data. For hypothesis 1 (RNT would decrease over the 10-day MBI training phase), a multilevel model was estimated on the RNT, as assessed by the EMA. We assumed a 2-level nested structure, with assessment-level variables nested within the person-level variables. The model is formulated as follows:

Prompt level:

*RNT_ij_* = *β*_0_*_j_* + *β*_1_*_j_Time_ij_* + *r_ij_* **(1)**

Person level:

*β*_0_*_j_* = *γ*_00_ + *γ*_01_*Group_j_* + *u*_0_*_j_* **(2)**

*β*_1_*_j_* = *γ*_10_ + *γ*_11_*Group_j_* + *u*_1_*_j_* **(3)**

*RNT_ij_* is the momentary level of RNT for participant *i* for the prompt *j* of the EMA. *Group* is a dummy code indicating group allocation, with 0 for the control group and 1 for the intervention group. The residual is denoted by *r*. The intercept and slopes (*β*_0_*_j_* and *β*_1_*_j_*) were allowed to vary across participants with person-level random effects (*u*_0_*_j_* and *u*_1_*_j_*), and the cross-level interaction effect (*γ*_11_) represents the group differences in the (linear) change over the MBI training phase. In other words, we tested whether the rate of change in RNT (*β*_1_*_j_*) would differ between the 2 groups, and this difference was modeled by the interaction effect *γ*_11_. We performed model selection using information criteria (with the ML estimator) to see whether a polynomial function would better approximate the changing pattern of RNT than a linear function of time. However, we found that the linear function was better than the quadratic and cubic functions, and thus we decided to focus exclusively on the linear change in the *Results* section.

To test the effects of our MBI at the T1 and T2 assessments (ie, hypothesis 2), we estimated a similar 2-level model for each of the 4 outcome measures (but with the restricted ML estimator): the PTQ and DASS depression, anxiety, and stress subscales. Each model is formulated as follows:

Assessment level:

*Outcome_ij_* = *β*_0_*_j_* + *β*_1_*_j_DT*1*_ij_* + *β*_2_*_j_DT*2*_ij_* + *r_ij_* **(4)**

Person level:

*β*_0_*_j_* = *γ*_00_ + *γ*_01_*Group_j_* + *u*_0_*_j_* **(5)**

*β*_1_*_j_* = *γ*_10_ + *γ*_11_*Group_j_* + *u*_1_*_j_* **(6)**

*β*_2_*_j_* = *γ*_20_ + *γ*_21_*Group_j_* + *u*_2_*_j_* **(7)**

*Outcome_ij_* is the outcome value of participant *i* at assessment *j*. The time variables *DT*1 and *DT*2 are dummy codes representing the differences between the T0 and T1 assessments and between the T0 and T2 assessments, respectively*.* Both the intercept and slopes (*β*_0_*_j_*, *β*_1_*_j_*, and *β*_2_*_j_*) were allowed to vary across participants with person-level random effects (*u*_0_*_j_*, *u*_1_*_j_*, and *u*_2_*_j_*, respectively). The variance of random effects was fixed at 0 if the estimates exceeded 0. The 2 interaction effects *γ*_11_ and *γ*_21_ were of particular interest because they represented group differences in the changes between the 2 given assessments.

Post hoc (simple slope) tests were conducted for any significant interaction effects between time and group. Effect sizes were defined in the framework of the growth-curve modeling analysis (GMA) [56], that is, *d*_GMA_ was calculated by first multiplying the fixed effect by time (ie, the average change in a given time window) and then dividing the product by the raw SD of the outcome. This metric is comparable with Cohen *d*. For each analysis, we tested whether the results were unchanged even after controlling for covariates (ie, age, gender, and spontaneous engagement in mindfulness practice up to the follow-up assessment).

## Results

### Descriptive Data and Compliance

The final sample consisted of 68 participants aged 18 to 55 (mean 26.79, SD 9.41) years (n=58, 85% women). The descriptive statistics for the T0, T1, and T2 assessments are presented in Tables 1 and 2. The mean compliance among the 2 groups during the EMA was 76.73% (SD 16%). Participants in the intervention group (35/68, 51%) had a slightly higher mean compliance (78%, SD 16%) than participants in the control group (31/68, 49%; 75%, SD 16%). Of the 31 participants in the control group, 2 (6%) had no available EMA data (n=1, 50% did not start the EMA, and n=1, 50% did not respond to any EMA signals). Participants showed excellent adherence to the intervention; they performed our 5-minute body scan for a mean 86.98% (SD 11%) of the days during the MBI training phase. Only 1 (3%) of the 35 participants performed <70% of the MBI training sessions. At the T2 assessment, of the 31 participants in the intervention group, 12 (39%) reported that they had stopped mindfulness practice after the 10-day training period, 18 (58%) had continued the practice at least once a month, and 1 (3%) had practiced mindfulness for most of the days. Note that the self-guided continuation of the MBI exercises was a spontaneous reaction because we did not instruct the participants to continue the practice. In the control group, of the 28 participants, 11 (39%) reported having engaged in spontaneous mindfulness practice after the T1 assessment, and 2 (7%) reported having practiced mindfulness on most of the days.

**Table 1:** Demographics and baseline characteristics (n=68).

Variable		Control group (n=33)	Intervention group (n=35)	Group difference		
				*t* test (*df*)	Chi-square (*df*)	*P* value
Age (years), mean (SD)		27.76 (10.69)	25.89 (8.07)	0.81 (66)	N/A^a^	.42
**Gender, n (%)**						
	Man	4 (12)	6 (17)	N/A	0.1 (1)	.81
	Woman	29 (88)	29 (83)	N/A	0.1 (1)	.81
EMA^b^ compliance (%), mean (SD)		78 (16)^c^	75 (16)	0.59 (64)	N/A	.55
**T0 (baseline) measures, mean (SD)**						
	PTQ^d^	31.48 (9.27)	28.97 (10.45)	1.05 (66)	N/A	.30
	DASS-D^e^	3.94 (3.57)	3.81 (3.96)	0.14 (63)	N/A	.89
	DASS-A^f^	3.48 (3.31)	2.69 (2.97)	1.05 (66)	N/A	.30
	DASS-S^g^	5.94 (4.04)	6.71 (3.91)	−0.78 (64)	N/A	.44

^a^N/A: not applicable.

^b^EMA: ecological momentary assessment.

^c^Of the 33 participants in the control group, 2 (6%) had no EMA data.

^d^PTQ: Perseverative Thinking Questionnaire.

^e^DASS-D: Depression Anxiety Stress Scale, depression subscale.

^f^DASS-A: Depression Anxiety Stress Scale, anxiety subscale.

^g^DASS-S: Depression Anxiety Stress Scale, stress subscale.

**Table 2:** Descriptives at the postintervention and follow-up assessments.

Variables	Postintervention assessment, mean (SD)		Follow-up assessment, mean (SD)	
	Control group (n=29)	Intervention group (n=34)	Control group (n=28)	Intervention group (n=31)
PTQ^a^	27.55 (11.33)	22.68 (8.79)	28.00 (9.66)	20.32 (8.56)
DASS-D^b^	3.19 (3.64)	2.52 (3.13)	3.39 (3.02)	2.65 (3.12)
DASS-A^c^	2.39 (2.77)	1.33 (2.03)	2.44 (2.55)	1.58 (2.20)
DASS-S^d^	5.59 (5.00)	3.88 (2.78)	5.22 (3.47)	4.35 (3.15)

^a^PTQ: Perseverative Thinking Questionnaire.

^b^DASS-D: Depression Anxiety Stress Scale, depression subscale.

^c^DASS-A: Depression Anxiety Stress Scale, anxiety subscale.

^d^DASS-S: Depression Anxiety Stress Scale, stress subscale.

### Hypothesis 1: EMA-Assessed RNT Throughout the MBI Training Phase

**Figure 3:**
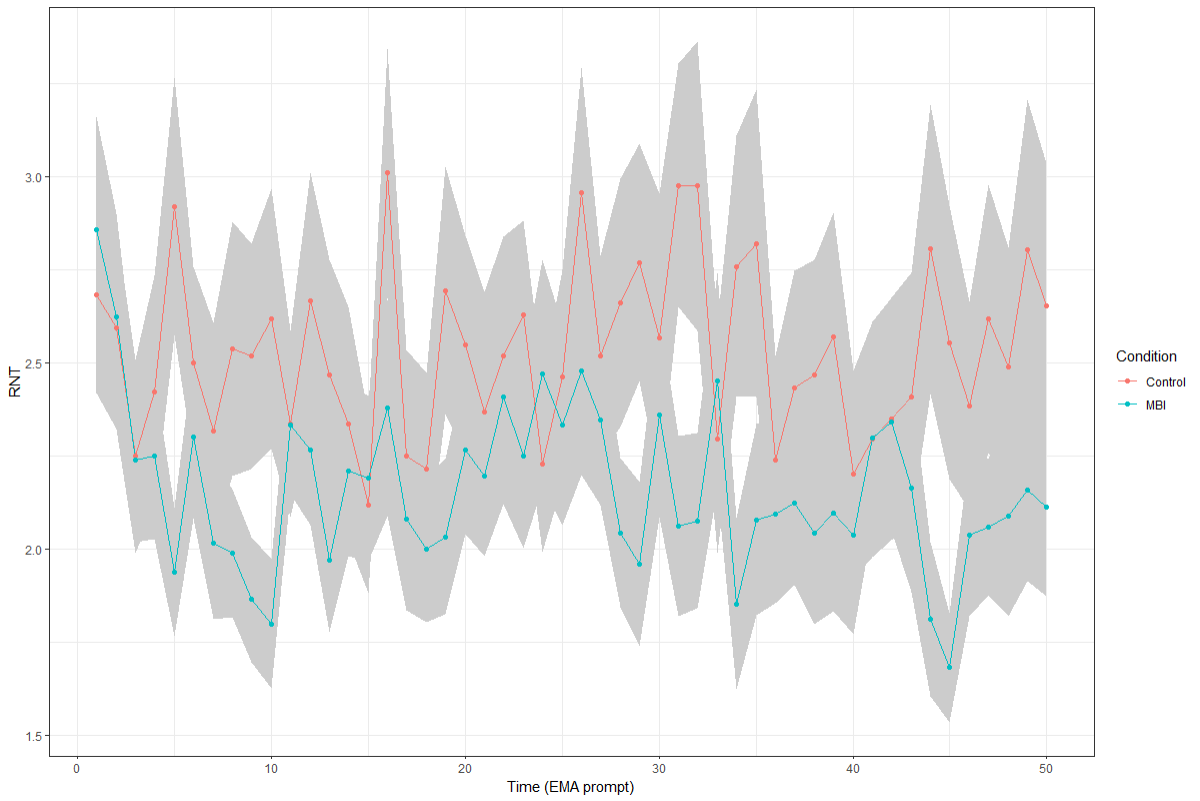
Development of repetitive negative thinking (RNT) over the course of the ecological momentary assessment (EMA). Mean RNT scores are illustrated for the no-training control group and the intervention group; the latter performed a 10-day 5-minute mindfulness-based intervention (MBI) at night. The figure covers the 10-day MBI training phase, during which EMA data were collected. The time spans 50 prompts across the 10-day EMA. The gray field indicates the SE.

To test the temporal changes in RNT throughout the MBI, a multilevel model was estimated with *group*, *time* (EMA prompt), and their interaction as predictors of EMA-assessed RNT (Table 3). The results showed no significant main or interaction effects, suggesting that the level of RNT did not change over time and that there was no significant group difference in the rate of change (Figure 3). As an exploratory analysis, we examined the effects of *group* and *time* on the levels of RNT reported after 8 PM. The results showed neither a significant main effect nor a significant interaction effect. It is unlikely that the MBI specifically reduces the evening levels of RNT. However, note that the last EMA signal was sent before 10 PM each day, whereas a body scan was performed right before going to sleep (typically after the last EMA signal was sent). Our design might be suboptimal to capture the immediate effect of a body scan on RNT at night.

**Table 3:** Multilevel model predicting ecological momentary assessment–assessed repetitive negative thinking^a^.

Predictor	*B* (95% CI; SE)	*t* test (*df*)	*P* value
Intercept	2.43 (2.08 to 2.77; 0.18)	13.84 (66.8)	<.001
Time^b^	0.00 (−0.01 to 0.01; 0.00)	0.61 (67.6)	.54
Group^c^	−0.17 (−0.64 to 0.31; 0.24)	−0.69 (66.7)	.49
Time:group	−0.01 (−0.02 to 0.00; 0.01)	−1.02 (67.1)	.31

^a^There were 2448 observations across 68 participants.

^b^Ecological momentary assessment prompt ranging from 1 to 50.

^c^Mindfulness-based intervention group versus no-training control group.

### Hypothesis 2: Effects From Pre- to Postintervention and Follow-Up Assessments

**Figure 4:**
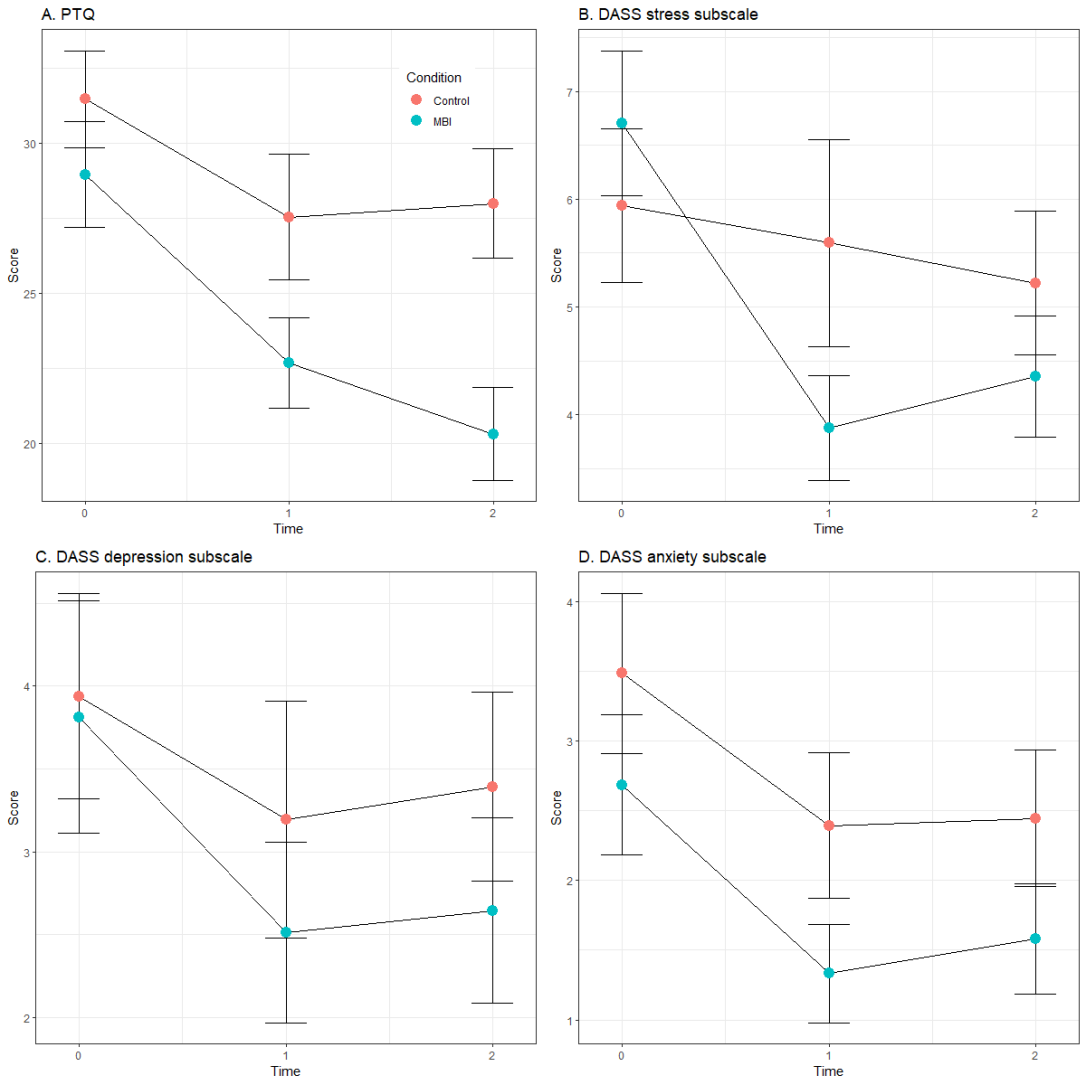
Changes in repetitive negative thinking and psychological distress (A: repetitive negative thinking, B: stress, C: depressive symptoms, and D: anxiety) from baseline to the postintervention and 2-month follow-up assessments. Changes are illustrated for the no-training control group and the intervention group; the latter performed a 10-day 5-minute mindfulness-based intervention (MBI) at night. The error bars indicate SEs. Time: 0=baseline assessment, 1=postintervention assessment, and 2=2-month follow-up assessment. DASS: Depression Anxiety Stress Scale; PTQ: Perseverative Thinking Questionnaire.

To test hypothesis 2, separate multilevel models were estimated for each outcome measure of the PTQ and the DASS. For the PTQ, the results (Table 4) showed significant main effects of time (both for the postintervention assessment [DT1] and the follow-up assessment [DT2]), qualified by a significant interaction between *group* and DT2 (but not DT1). Both groups exhibited an equally significant reduction from the T0 to T1 assessments (intervention group: *B*=−6.40, SE 1.29; *t*_57.2_=*−*4.98; *P*<.001; *d*_GMA_=−0.66; control group: *B*=−4.18). However, the intervention group showed a larger reduction from the T0 to T2 assessments than the control group (intervention group: *B*=−8.70, SE 1.35; *t*_95.4_=−6.43; *P*<.001; *d*_GMA_=−0.91; control group: *B*=−3.31). These results suggest that the PTQ score continued to decrease even after the completion of the MBI (Figure 4A). The results were overall unchanged even after controlling for age, gender, and engagement in mindfulness practice, which spontaneously continued up to the follow-up assessment. As an exploratory analysis, we tested the mean differences in the PTQ scores at the follow-up assessment between participants who engaged in mindfulness practice and those who did not. We identified no significant difference for the MBI group (mean 21.6, SD 9.45; mean 18.3, SD 6.80; *t*_29_=−1.07; *P*=.29). However, for the control group, participants who spontaneously performed mindfulness practice after the T1 assessment up to the follow-up assessment showed lower PTQ scores than those who did not (mean 22.36, SD 8.35; mean 31.64, SD 8.82; *t*_26_=2.78; *P*=.01).

For the DASS, we found no significant main or interaction effects on the depression and anxiety subscales (Table 4). However, the stress subscale had significant interactions between group and the 2 time dummies, suggesting that the intervention group experienced a larger reduction in stress from the T0 to T1 assessments (*B*=−2.65, SE 0.55; *t*_89.6_=−4.80; *P*<.001; *d*_GMA_=−0.78) and from the T0 to T2 assessments (*B*=−2.11, SE 0.55; *t*_70.9_=−3.83; *P*<.001; *d*_GMA_=−0.60) than the control group (T0 to T1 assessments: *B*=−0.05; T0 to T2 assessments: *B*=−0.49; Figures 4B-4D). We added age and gender as covariates to the model, which did not change the results. Another covariate, namely continued engagement in mindfulness up to the follow-up assessment, had no substantial influence on the interaction between group and DT1 (*B*=−1.90, SE 0.82; *t*_108.3_=−2.33; *P*=.02), but this covariate reduced the effect slightly at DT2 (*B*=−1.33, SE 0.80; *t*_108.3_=−1.66; *P*=.01). This may point to the possibility that the sustained stress reduction at the follow-up assessment is attributed to the spontaneous engagement in mindfulness practice after the postintervention assessment. However, an exploratory analysis identified no significant difference in the levels of stress at the follow-up assessment between participants who continued mindfulness practice and those who did not (mean 4.63, SD 3.53; mean 3.92, SD 2.50; *t*_29_=0.61; *P*=.55) for the MBI group. For the control group, participants who spontaneously performed mindfulness practice after the T1 assessment up to the follow-up assessment showed slightly lower stress levels than those who did not (mean 3.60, SD 2.07; mean 6.18, SD 3.81; *t*_25_=1.96; *P*=.06).

**Table 4:** Multilevel models predicting the effect of a 5-minute mindfulness-based intervention at nighttime on repetitive negative thinking and psychological distress symptoms (n=68)^a^.

Outcome and predictor			*B* (95% CI; SE)		*t* test (*df*)		*P* value
**PTQ^b^**							
	Intercept	31.48 (28.19 to 34.78; 1.68)		18.74 (96.0)		<.001	
	DT1^c^	−4.18 (−6.88 to −1.48; 1.38)		−3.04 (59.0)		.002	
	DT2^d^	−3.31 (−6.09 to −0.53; 1.42)		−2.33 (94.8)		.02	
	Group	−2.51 (−7.10 to 2.08; 2.34)		−1.07 (96.0)		.28	
	Group^e^:DT1	−2.21 (−5.91 to 1.48; 1.88)		−1.18 (58.2)		.24	
	Group:DT2	−5.39 (−9.24 to −1.55; 1.96)		−2.75 (95.1)		.006	
**DASS-D^f^**							
	Intercept	3.94 (2.76 to 5.12; 0.60)		6.53 (99.6)		<.001	
	DT1	−0.56 (−1.64 to 0.51; 0.55)		−1.03 (113.4)		.30	
	DT2	−0.46 (−1.50 to 0.58; 0.53)		−0.86 (112.7)		.39	
	Group	−0.19 (−1.86 to 1.47; 0.85)		−0.23 (102.9)		.82	
	Group:DT1	−0.58 (−2.05 to 0.89; 0.75)		−0.77 (113.2)		.44	
	Group:DT2	−0.41 (−1.88 to 1.05; 0.75)		−0.55 (113.2)		.58	
**DASS-A^g^**							
	Intercept	3.48 (2.55 to 4.42; 0.48)		7.30 (97.4)		<.001	
	DT1	−1.00 (−1.81 to −0.18; 0.42)		−2.39 (114.7)		.02	
	DT2	−0.72 (−1.55 to 0.11; 0.42)		−1.71 (115.1)		.09	
	Group	−0.80 (−2.10 to 0.51; 0.67)		−1.20 (97.4)		.23	
	Group:DT1	−0.26 (−1.38 to 0.86; 0.57)		−0.46 (114.2)		.65	
	Group:DT2	−0.25 (−1.39 to 0.89; 0.58)		−0.43 (114.7)		.67	
**DASS-S^h^ (n=67)**							
	Intercept	5.94 (4.64 to 7.24; 0.66)		9.00 (89.3)		<.001	
	DT1	−0.05 (−1.22 to 1.11; 0.59)		−0.09 (84.5)		.93	
	DT2	−0.49 (−1.63 to 0.65; 0.58)		−0.85 (71.1)		.40	
	Group	0.70 (−1.09 to 2.50; 0.92)		0.77 (90.1)		.44	
	Group:DT1	−2.59 (−4.18 to −1.01; 0.81)		−3.20 (86.9)		.001	
	Group:DT2	−1.62 (−3.19 to −0.05; 0.80)		−2.02 (71.0)		.04	

^a^There were 184 to 190 observations across participants.

^b^PTQ: Perseverative Thinking Questionnaire.

^c^DT1: Dummy-coded time variables representing the differences between the T0 (baseline) and T1 (day after the intervention) assessments.

^d^DT2: Dummy-coded time variables representing the differences between the T0 (baseline) and T2 (2-month follow-up) assessments.

^e^Mindfulness-based intervention group versus no-training control group.

^f^DASS-D: Depression Anxiety Stress Scale, depression subscale.

^g^DASS-A: Depression Anxiety Stress Scale, anxiety subscale.

^h^DASS-S: Depression Anxiety Stress Scale, stress subscale.

## Discussion

### Principal Findings

Brief MBIs are a promising tool for improving mental health in an accessible way. However, little is known about the real-time effect of minimal (ie, 5-minute) MBIs on RNT, which is assumed to be the underlying reason for the effectiveness of an MBI. This study investigated whether a minimal MBI (a 5-minute body scan performed daily for 10 days) before sleeping would have positive effects on RNT and psychological distress symptoms (ie, depressive, anxiety, and stress symptoms) by comparing an intervention group with a no-training control group. First, we examined RNT in real time using EMA during the MBI training phase (hypothesis 1). Contrary to our expectations, the results showed no significant group differences in RNT during the training phase. Second, we examined the effects of the 5-minute body scan immediately after the intervention (T1) and at the 2-month follow-up assessment (T2; hypothesis 2). We found that both groups had significant reductions in questionnaire-based RNT at the postintervention assessment (although there was no group difference); however, the intervention group showed a significantly larger reduction than the control group at the follow-up assessment. As hypothesized, only the intervention group showed a decrease in stress at both postintervention and 2-month follow-up time points. We did not find any intervention effects on depressive or anxiety symptoms.

Taking a closer look at hypothesis 1, the results regarding the momentary levels of RNT, as assessed by EMA, showed that a minimal MBI may not induce acute changes in RNT during the training phase. This finding is inconsistent with the theory [9] and contradicts previous empirical evidence where RNT declined rapidly during the first week of an MBI [25]. However, given that the previous EMA study [25] used more intensive training (ie, a 20-minute daily MBI), it is possible that our 5-minute body scan was not sufficient to observe immediate changes in RNT.

Examining hypothesis 2, we found that a minimal MBI administered before sleeping is effective in decreasing questionnaire-based RNT, although significant group differences emerged only at the T2 assessment and not at the T1 assessment. Although the effect of the intervention started to appear early, it was only after continued improvement during the follow-up phase that the questionnaire-based RNT scores were significantly different from the control condition, that is, the effect seems to have accumulated gradually, and the difference from the control condition became visible not immediately during and after the intervention but at the 2-month follow-up assessment with increased mindfulness practice experience. This explanation is supported by the fact that 61% (19/31) of the participants in the intervention group continued mindfulness practice even after the MBI training phase. However, the exploratory analysis revealed no statistically significant difference in RNT levels at the T2 assessment between participants who continued the MBI and those who did not, which contradicts the idea that the MBI effect accumulates through spontaneous practice. Another aspect that should be mentioned is that the control group showed a significant decrease in RNT at the postintervention assessment. This decrease may suggest that the EMA worked as a self-monitoring technique, which helped participants to realize how frequently they had engaged in RNT. In addition, control participants who spontaneously started mindfulness practice after the T1 assessment showed lower levels of RNT and stress than those who did not. Taken together, it seems that the spontaneous use of mindfulness practice plays an important role to maintain and enhance the effect of mindfulness practice, but we cannot draw a solid conclusion only from these data. Furthermore, because a previous EMA study [25] documented a second significant decline in RNT between the 30th and 42nd day of mindfulness practice, it may be interesting to extend the active training phase of the 5-minute MBI, depending upon participant compliance.

Examining psychological distress symptoms, we found that participants in the intervention group showed significantly larger decreases in their stress levels than those in the control group at both the T1 and T2 assessments. This finding is in line with the body of evidence reporting the beneficial effects of an MBI on stress [12,13,57]. However, the effect of our minimal MBI was not significant for depressive or anxiety symptoms. Given that our sample showed low levels of depressive and anxiety symptoms at baseline, our minimal MBI sought to diminish what had already been low, which could be responsible for the nonsignificant effects on the depressive and anxiety outcomes (Table 1). Our sample only showed slightly higher means of the DASS depression and anxiety subscales scores than those of a large nonclinical sample in the literature: 2.83 for depressive symptoms, 1.88 for anxiety symptoms, and 4.73 for stress symptoms [58]. By contrast, the means of DASS scores in a clinical sample were 6.3 for depressive symptoms, 4.1 for anxiety symptoms, and 5.7 for stress symptoms [48]. Future studies may benefit from recruiting participants with a broader range of subclinical psychological distress symptoms.

Moreover, stress may be more acutely influenced by mindfulness practice than depressive and anxiety symptoms via autonomic arousal: in a study measuring heart rate variability over a 10-day MBI, perceived stress and acute physiological stress decreased for the mindfulness practice [57]. By contrast, long-term cognitive changes may be required to alleviate depressive and anxiety symptoms. On the one hand, more practice may be needed for cognitive change because, initially, awareness is brought to unpleasant experiences, and it is only in the second step that coping takes place through the acceptance of these experiences and the development of a nonjudgmental attitude [6]. On the other hand, a different type of MBI may be needed to trigger cognitive changes because there is evidence showing that the mindfulness practice used in this study (ie, body scan) is inferior to other mindfulness practices (eg, sitting meditation and yoga) in its ability to enhance a nonjudgmental stance [59], and programs such as MBCT combine mindfulness meditation and cognitive techniques to elicit cognitive change (compare with the study by Segal et al [9]). Nonetheless, regarding the observed effects on RNT and stress, our findings are promising, bearing in mind that RNT is a target for the prevention and treatment of highly prevalent disorders such as depression and anxiety [27], which are considered a consequence of ongoing stress [12].

We observed excellent compliance rates for MBI completion in this study, underscoring the feasibility of our 5-minute body scan. In addition, we noticed continued interest in training in our sample because the majority of the participants (19/31, 61%) spontaneously maintained mindfulness practice after the active training phase. Thus, our data suggest that a 5-minute MBI at night is feasible and that the minimal time investment of 5 minutes can be seen as getting a foot in the door of mindfulness practice, leaving room for the possibility that those who experience benefits may eventually increase their practice times.

Similar to the findings of other studies in the field, our findings need to be interpreted considering a number of limitations. First, we did not use a blinded experimenter, and participants in both groups were informed about the aim of the study. Therefore, we cannot exclude the possibility that the results are biased by social desirability and a tendency to please the experimenter. This may explain the frequent (and somewhat unexpected) engagements in mindfulness practice among control participants, who might have been primed by the study information and assessments. Second, our data had an imbalance in terms of gender because the majority of the participants (58/68, 85%) in our study were women. Another potential issue concerning the sample characteristics is that we did not selectively recruit individuals with high levels of RNT or psychological distress. This is because we wanted to show the effectiveness of a minimal MBI used daily in a nonclinical population as a method of RNT reduction and stress management. However, the downside is that the effect might be underestimated because many of the participants showed low-to-moderate levels of RNT and distress at baseline. In addition, it should be noted that the sample size was relatively small (refer to the power analysis details in the *Participants* subsection of the *Methods* section), and this may have prevented us from detecting a significant effect of the MBI on EMA-assessed RNT. Third, adherence to the MBI was only measured via self-report, leaving the possibility that the actual amount of mindfulness practice differed from reported adherence. Fourth, we chose to implement an MBI at night; however, we were unable to incorporate conditions examining the effect of the MBI at other times of the day. Therefore, the opposite of our proposed rationale may be true. Although we have argued that targeting RNT at night when it is at its highest [33,34] would increase MBI effectiveness, it is possible that it was in fact harder to disengage from RNT at that time point. Thus, an MBI might be especially beneficial when one is in a state of mind where the attentional mindful focus can be attained throughout the practice without having to fight RNT. Fifth and last, we used a no-training control group, which provides relatively weak evidence for the efficacy of the intervention compared with an active control group.

### Conclusions

Notwithstanding the aforementioned limitations, the results of this study suggest that a 5-minute body scan before sleeping can effectively decrease RNT and stress. This minimal dosage makes an MBI more accessible than the traditional or full-package program to a wide range of audiences and would therefore be particularly suited for treating subclinical RNT and stress because it does not cost too much effort for training. However, future research may benefit from a more rigorous study design comparing the effects of different mindfulness exercises (eg, body scan, sitting meditation, and yoga) at different times of the day (eg, morning, noon, and night) to establish concrete recommendations for individual practitioners. This would enable the optimization of an MBI that could effectively promote mental health both acutely and in the long term.

## Abbreviations

DASS: Depression Anxiety Stress Scale
EMA: ecological momentary assessment
GMA: growth-curve modeling analysis
MBCT: mindfulness-based cognitive therapy
MBI: mindfulness-based intervention
MBSR: mindfulness-based stress reduction
ML: maximum likelihood
PTQ: Perseverative Thinking Questionnaire
RNT: repetitive negative thinking
